# Dentition and Its Derangements

**Published:** 1862-07

**Authors:** A. Jacobi


Dentition and its Derangements.—*By A. Jacobi*, M.D. *LECTURE IX*—Part 2.—Of the cutaneous affections mentioned, *erythema* is a very common occurrence. It consists of a superficial hyperæmia of the cutis, sometimes complicated with a small amount of exudation. According to the amount of serum transuding through the blood-vessels, more or less desquamation, in very small scales, will take place, but it forms no necessary part of the affection; at all events no formation of vesicles is observed. One of the characteristics is found in the gradual transition of the healthy into the erythematous surface. The etiology of erythema is simple enough; in a large number of cases the causes are: high temperature, and chemical, physical and mechanical irritation. Different names have been invented to suit the different forms and causes of the affection. They have called it *intertrigo*, when the case was one of erythema, produced by friction of two adjacent surfaces, near the folds of the femora, or neck, behind the ears, or near the mammæ of fat women. *Decubitus* is called the erythematous discoloraton of the skin depending on long-continued pressure over the sacrum, trochanter, etc. in protracted diseases. *E. lœve* it has been named when the consequence of considerable expansion of the skin in dropsy, or the result of local injuries. And a very common form is that depending on the irritation kept up on several places by discharges from the neighboring organs, thus from the nose on the upper lip and cheeks, from the bladder on penis, prepuce, scrotum, and femur.

Besides, erythema is regarded as having been observed epidemically, and with cyclical course. Whether this is founded on truth, or whether or not mild cases of scarlatina or measles were mistaken for a typical erythema, is an undecided question. At all events, the differential diagnosis between the several forms is sometimes a difficult one, and especially while both of the mentioned forms of epidemic diseases are frequently observed.

This uncertainty of a distinct diagnosis is not an uncommon thing. It proves that the same anatomical alteration in the skin takes place under different epidemic influences, usually modified by the nature of the latter. Thus, for instance, there is a difference of opinion, up to this day, on the occurrence of epidemic *roseola* or *rubeola*. While some positively deny its existence, taking it as a modified form of scarlatina, or measles, or erythema; others assume its occurrence as an independent disease, taking its own course like other epidemic affections, and not at all identical with measles or scarlatina. It is said to consist of isolated, irregular spots, usually with no vascular reaction, but sometimes attended with erethic, and even inflammatory fever; to be uncomplicated in the majority of cases, sometimes, however, to be attended with a tracheal cough, and thus mistaken for measles; sometimes with angina, and mistaken for scarlatina. Neither measles nor scarlatina are said to procure an immunity from rubeola, or *vice versa*, while its causes, regular or irregular migration, propagation, contagiousness or non-contagiousness, etc., remain open questions. Now these differences of opinion prove nothing else but the anatomical similarity between the large number of hyperæmic and inflammatory diseases of the skin, and the correctness of Dr. Simon's classification, and its foundation on anatomical principles.

*Strophulus* (*lichen* in adults) is generally laid down as the most common cutaneous affection during dentition. The exudation does not take place superficially, but into the cutis, sometimes with tumefaction of its tissue. Therefore no vesicles, but little nods, are formed. Its causes are frequently unknown. Whatever is apt to produce a single superficial erythema may just as well give rise to an affection of the cutis itself, in consequence of protracted or more intense injury; therefore coarse linen or flannel, dirt, animal parasites, and high temperature, are best known among its prominent causes. Some forms of "prickly heat" are simply strophulus. It is found either in groups, or isolated, or diffuse, or red, or normal, or extremely pale color, the latter depending on compression of neighboring blood-vessels. Simple strophulus takes a week or two to run its complete course with desquamation and full recovery, chronic cases being rare exceptions. A single form only, lichen *agrius*, which is not at all common in young children, is observed to be attended with severe itching, hyperæmia, and fever, and to be transformed in many cases into a severe form of eczema, with repeated attacks, and thickened and rigid condition of the skin. Simple strophulus requires no treatment, lichen agrius only the local application of cold and the administration of purgative baths, salves of tar and potassa, and the internal use of arsenic. I will add the single remark, that this latter remedy ought nowhere to be used except where the cutaneous affection goes along with a great deal of infiltration and induration; in these latter cases it is invaluable.

Caillault, one of the latest authors on cutaneous diseases, comprehends under the head of strophulus, all the erythematous, papular, vesicular, and postular forms of cutaneous affections occurring during the period of dentition, which disappear in a short time, prove very itching during their course, are observed on any part of the surface, and frequently complicated with intestinal affections. Its eruption is said to be often complicated with fever, which, if true, is easily explained by either the complication or the local irritation and itching. He is not at all particular concerning the memory of his unfortunate readers; the popular strophulus alone being subdivided by him into the forms of strophulus *intertinctus*, and *albidus*, and *candidus*, and *volaticus*, and *confertus*. These subdivisions may be justified as proofs of philological learning, other authors having availed themselves of the same, or similar names,before his time, but there is one fact on which not enough stress can be laid, viz., the occurrence of this form of cutaneous hyperæmia and exudation, not only during the period of dentition, but also before and afterwards. You will find a great many cases in even new-born children, whose skin is particularly irritable.

As to *herpes*, which is an acute and typical disease, when observed in children, the assumption of its being a symptom of dentition is not sustained by anything. It is observed in a large number of feverish diseases, especially in intestinal affections, pneumonia, and intermittent fever; the vesicles raised are very small, superficial, surrounded by a red areola, and always found in groups.

*Urticaria* (hives) consists of flat and large elevations, mostly of the color of the skin. It depends on serous infiltration of the papillary layer of the cutis, and is mostly an acute affection. Its causes are in no direct connection with dentition, adults suffering from the same affection when laboring under gastric disorders; and females, when affected with irritation of the uterus during pregnancy, menstruation, and uterine diseases, or even during the presence of a pessary in the vagina. Other causes are: local irritation by scratching, nettles, rhus toxicodendron and other euphorbiaceæ, some caterpillars and mollusca, fleas, mosquitoes, etc., the eating of strawberries, mushrooms, etc., in some persons, the use of copaiva; the presence of acute gastric catarrh from whatever cause, this latter giving the impression of a severe disease. But not the protrusion of a tooth. Nor does it appear that between urticaria as found in children, and again in adults, there is any remarkable difference, either in its external form and symptoms, or in its etiology.

It is true, as a general rule, that there are peculiarities in the diseases of the infantile skin, just as well as of other organs, and that the difference of ages even during infancy and childhood gives rise to a number of modifications in the form, color, etc., of cutaneous affections. But the pathological process itself is the very same, with the exception of the larger number of cutaneous affections in those ages, and of individual differences depending on either more or less irritability and impressibility of both system and skin. By this fact we are prevented from favoring the old assumption, lately again advocated by Caillault, of "diathesis" being the cause of the differences as to the forms and natures of cutaneous affections. At the same time we are enabled to answer the questions laid before you in the course of this lecture in the following manner—that there is, between the protrusion of a tooth, and the appearance of the larger number of cutaneous diseases, no direct relation of causality; that they do not show themselves with the swelling of the gums; that they do not disappear with or after the final protrusion of a tooth or a group of teeth, and that they do not return with the renewed attempts of another tooth, or group of teeth, to break through the gums. Only those forms of cutaneous diseases scarcely deserving the name, which depend on an occasional hyperæmia of the surface, brought on by general feverish irritation, or the physiological injection of the blood-vessels of the head about this time, are observed during the periods of dentition. They, and the absence of correct diagnoses generally, and the frequency of skin affections in this early period of life, are the reasons of the long continuance of the old assumption of a connection between external diseases and dentition.

To what extent this is true, I shall finally show by some more extensive remarks on two cutaneous affections very common in infantile age, viz., eczema and impetigo. They are deserving of our particular attention, for their frequent occurrence, the difficulty in removing them, their frequent returns when cured, and their real or assumed connection with the physiclogical and pathological condition of the teeth, brain and system.—*American Medical Times.*

---

The present war is likely te contribute in many ways to our knowledge of the diseases and injuries incident to warfare under the peculiar circumstances in which our troops are placed. The influences of climate, season, character of the food, nature of the clothing, etc., will all come under the eye of numerous competent medical observers, and we look for very interesting and valuable results, when the sum of this extended range of observation shall have been consolidated in a condensed, practical form. We are glad to see that the government has taken the initial steps to secure the collecting together all the recorded results of the experience of all our army surgeons for this very purpose. A great deal of valuable observation must undoubtedly go unrecorded, except as it shall appear hereafter in the writings of men whose memory shall furnish a daguerreotype of what their hands were too busy at the time to take note of. So overworked have been the army surgeons and those in charge of the extemporized hospitals in the neighborhood of our large armies, that it could not be expected that any proper record of professional experience from day to day could always be kept. Nevertheless, there must be much that will be gathered up for the guidance and instruction of those to come after us.

Among other important results we hope to have very valuable contributions to our knowledge of the effects of anæsthetics, as administered in their various forms and combinations, the comparative safety with which different agents may be employed, the precautions to be taken to secure immunity from the fatality which has in civil life so frequently made chloroform a doubtful blessing, and the most efficacious means to neutralize the alarming symptoms so often produced by it. From the formula of supplies furnished to army surgeons by our government, as given in Dr. Tripler's handbook, we gather that chloroform is the only anæsthetic placed in their hands. Sulphuric ether has been given by State governments in some instances, we believe, to the regiments from their own States, and we hear of both chloroform and sulphuric ether being used in some of the hospitals of the Peninsula. We sincerely hope that we shall have honest, unprejudiced reports of the observed results of the use of these agents. If the statements of European authorities as to the complete safety with which chloroform was employed in the Crimean war are to be relied upon, sulphuric ether may at once be given up as altogether a superfluity. But we confess we can not believe these statements in their wholesale assertions. There must have been many instances of unreported deaths in the field or in the trenches, where the very circumstances of the case made it absolutely impossible to record the fact that chloroform was the fatal agent; without taking into account the indifference or the reticence of those who would not take the trouble or did not wish to preserve evidence of so disagreeable a character.

In the interesting work of M. Baudens, Medical Inspector of the French army in Corsica, Italy and the Crimea, just published in an English form by Baillière Brothers, New York, are some interesting remarks on this subject. Some of our Boston readers, by the way, will remember the statements made here not long since, with a good deal of confidence, that the French army in the Crimea used a mixture of chloroform and sulphuric ether for anæsthetic purposes. M. Baudens sets that question at rest. It is somewhat amusing to observe how ready European writers are to keep out of sight the fact that the use of anæsthetics in surgery is an American discovery, and to attach to the employment of a particular agent the whole value of this great blessing. At the same time that France gives credit to one of her sons for it, Great Britain recognizes Prof. Simpson as the great discoverer of anæsthetics as represented by chloroform; while the great fact, which lies at the basis of the application of all such agents, is without doubt an American discovery. It is note-worthy, too, that chloroform itself was made in the United States by an independent experimenter at about the same time that it was in Europe. The following are some of the passages relating to this subject in M. Baudens' valuable work, which we propose to notice more fully hereafter:

"In the Crimean war, surgical science was first aided by a recent discovery due to the researches of M. Fleurens, and which until then had not been used on the battle-field. We allude to the anæsthetic action of chloroform, whose wonderful effect in relieving the terrible pains of the wounded has been often useful in healing their wounds. Its use permits us to trim wounds, mortal in appearance, which the surgeon would not have ventured to treat with so much energy, for fear of exciting new sufferings. These wounds, being thus treated, become less painful, and sometimes we are astonished at their unexpected cure. * * * *

"By subduing pain, chloroform gives a calmness and mental tranquillity to the wounded, very favorable to healing. It deprives the traumatic fever of an excess of reaction, often caused by the anxiety of the patient. Before the discovery of this precious agent, some soldiers, it is true, endured amputations without a groan. I have seen an Arab continue to smoke his pipe, while I took off his arm; but this paroxysm of courage, gained by a great exaltation, fell some days after into a nervous depression, so much the more dangerous on account of the stoicism which had sustained him. * * *

"We know that, in great operations, death ensues oftener from nervous prostration, consequent upon excess of suffering, than from hemorrhage. It is the same with animals. M. Claude Bernard observed that, in laying open the spinal column, in experiments upon rabbits, they always died at once, unless they had previously been rendered insensible by chloroform.

"But used imprudently, this agent, while it takes away suffering, may also take away life. It shares this sad privilege with the most potent remedies; taken in large doses, they are for the most part poisonous, and kill instead of curing. The danger may perhaps be certainly avoided, by observing certain rules, and especially by not pressing the inhalation to its extreme limit. This extreme limit is, in my opinion, when in accordance with the precept laid down for several years, we pass the stage of *insensibility* and reach that of *collapse*, and a complete muscular prostration. This condition is reached when a limb is lifted up and falls like a mass of dead matter; for then life almost borders upon death, it has retired to the vital center, placed by M. Fleurens in the medulla oblongata, at the origin of the eighth pair of nerves, which absolutely control the function of the heart and lungs. To approach this point is rash temerity; to reach it is death. * * * The physicians of the army of the East were of this opinion, and administered chloroform with great prudence, stopping at the point of insensibility, and never intentionally exceeding it. They had, therefore, no fatal accidents to deplore, although in the campaign of the East this agent was employed at least thirty thousand times. In the Crimea alone, it was administered to more than twenty thousand wounded, according to the estimate of M Scrive. The physicians of the Sardinian army, at the beginning of the campaign, hesitated to use it, but the success of our surgeons soon gave them confidence in its efficacy. Henceforward we may have a steadfast confidence in chloroform, and thank Providence for having allowed human skill to invent an agent that can suspend pain."*—*Boston Med. and Surg. Journal.*

---

Creosote Collodion.—As an antisceptic, Mr. Stanislaus Martin employs a combination of fifteen parts by weight of creosote and ten of collodion. The solution is of a glutinous consistency.—*Am. Drug. Circular and Chem. Gaz.*

---

* The English surgeons, in the Crimea, employed chloroform very generally, and McLeod considered their confidence in its efficacy greater than among the French. One well established fatal case occurred from its use, while a great number of operations were successfuily performed, which would have otherwise not been attempted. The *morale* of the wounded was better sustained, and the courage and comfort of the surgeons increased. Its moderate use was not found to result in depression, but on the contrary, it often supported the strength of the patient uuder the operation, and it was never more successful than when used immediately after the injury, before the constitution had begun to suffer from the nervous irritation liable to follow a wound. It acts more rapidly upon persons who have lost much blood, and hence more care is necessary in such cases.

